# The Effects of a Combination of Ion Channel Inhibitors in Female Rats Following Repeated Mild Traumatic Brain Injury

**DOI:** 10.3390/ijms19113408

**Published:** 2018-10-31

**Authors:** Yilin Mao, Anna M. B. Black, Hannah R. Milbourn, Samra Krakonja, Michael Nesbit, Carole A. Bartlett, Brooke Fehily, Ryu Takechi, Nathanael J. Yates, Melinda Fitzgerald

**Affiliations:** 1 Curtin Health Innovation Research Institute, Faculty of Health Sciences, Curtin University, Kent St, Bentley, WA 6102, Australia; yilin.mao@curtin.edu.au (Y.M.); ab2458@bath.ac.uk (A.M.B.B.); michael.nesbit@curtin.edu.au (M.N.); r.takechi@curtin.edu.au (R.T.); 2 Perron Institute for Neurological and Translational Science, Sarich Neuroscience Research Institute Building, 8 Verdun St, Nedlands, WA 6009, Australia; 3 Experimental and Regenerative Neurosciences, School of Biological Sciences, The University of Western Australia, 35 Stirling Hwy, Crawley, WA 6009, Australia; hannah.milbourn@bath.edu (H.R.M.); 21161317@student.uwa.edu.au (S.K.); carole.bartlett@uwa.edu.au (C.A.B.); brooke.fehily@uwa.edu.au (B.F.); nathanael.yates@uwa.edu.au (N.J.Y.); 4 School of Human Sciences, The University of Western Australia, 35 Stirling Hwy, Crawley, WA 6009, Australia

**Keywords:** neurotrauma, repeated mild traumatic brain injury, ion channel inhibitors, oxidative stress, node of Ranvier

## Abstract

Following mild traumatic brain injury (mTBI), the ionic homeostasis of the central nervous system (CNS) becomes imbalanced. Excess Ca^2+^ influx into cells triggers molecular cascades, which result in detrimental effects. The authors assessed the effects of a combination of ion channel inhibitors (ICI) following repeated mTBI (rmTBI). Adult female rats were subjected to two rmTBI weight-drop injuries 24 h apart, sham procedures (sham), or no procedures (normal). Lomerizine, which inhibits voltage-gated calcium channels, was administered orally twice daily, whereas YM872 and Brilliant Blue G, inhibiting α-amino-3-hydroxy-5-methyl-4-isoxazolepropionic acid (AMPA) and P2X_7_ receptors, respectively, were delivered intraperitoneally every 48 h post-injury. Vehicle treatment controls were included for rmTBI, sham, and normal groups. At 11 days following rmTBI, there was a significant increase in the time taken to cross the 3 cm beam, as a sub-analysis of neurological severity score (NSS) assessments, compared with the normal control (*p* < 0.05), and a significant decrease in learning-associated improvement in rmTBI in Morris water maze (MWM) trials relative to the sham (*p* < 0.05). ICI-treated rmTBI animals were not different to sham, normal controls, or rmTBI treated with vehicle in all neurological severity score and Morris water maze assessments (*p* > 0.05). rmTBI resulted in increases in microglial cell density, antioxidant responses (manganese-dependent superoxide dismutase (MnSOD) immunoreactivity), and alterations to node of Ranvier structure. ICI treatment decreased microglial density, MnSOD immunoreactivity, and abnormalities of the node of Ranvier compared with vehicle controls (*p* < 0.01). The authors’ findings demonstrate the beneficial effects of the combinatorial ICI treatment on day 11 post-rmTBI, suggesting an attractive therapeutic strategy against the damage induced by excess Ca^2+^ following rmTBI.

## 1. Introduction

Mild traumatic brain injury (mTBI), also known as concussion, has been increasingly recognised as a public health issue, because repeated injuries may result in exacerbated and persisting post-concussive syndrome. [1,2,3]. Following injury to the central nervous system (CNS), high concentrations of the neurotransmitter glutamate are released from damaged neurons and activate *N*-methyl-d-aspartate (NMDA) ionic channels and α-amino-3-hydroxy-5-methyl-4-isoxazolepropionic acid (AMPA) receptors on surrounding neurons and glial cells [4]. The efflux of potassium ions (K^+^) depletes intracellular K^+^ reservoirs via the activated NMDA and AMPA receptors and voltage-gated potassium channels [4,5], whereas the uncontrolled influx of calcium ions (Ca^2+^) enters the cells via a number of ion channels, including but not limited to: voltage-gated calcium channels (VGCCs) [6,7], AMPA receptors [8,9] and ionotropic P2X_7_ receptors [10,11]. To restore the ionic balance, the activity of ATP-dependent sodium/potassium ion pumps (Na^+^/K^+^-ATPase) is increased, requiring a high level of glucose metabolism [12]. However, the activation of Na^+^/K^+^-ATPase rapidly reduces intracellular energy stores and causes neurons to produce more energy in a quick but inefficient way, namely, glycolysis [13]. Concurrently, oxidative metabolism is disrupted to overproduce reactive oxygen and nitrogen species (ROS and RNS, respectively) [12,14] and increase mitochondrial permeability [15,16,17], leading to further loss of neurons and associated functions. Therefore, ion channels are a candidate target for therapeutic intervention.

When excessive amounts of ROS and RNS overcome innate antioxidant capacity, oxidative stress arises, associated with oxidation of cellular components, as shown in several rodent models of mTBI [18,19,20,21,22,23]. Oxidative stress can induce DNA-protein and DNA double strand breaks, crosslinking, and oxidative modification of bases [24]. Altered levels of DNA damage and manganese-dependent superoxide dismutase (MnSOD), an innate antioxidant, have been reported in several mouse models of mTBI [25,26,27,28], but not acutely following repeated mild traumatic brain injury (rmTBI) in rats [23]. Oxidative damage to the lipid content of brain tissue was observed as early as day 4 following rmTBI in the authors’ previous study [23]. As a consequence of oxidative damage, compromised cellular machinery initiates apoptosis of neurons and glia [29,30,31,32]. The nodes of Ranvier, with a high concentration of ion channels, have been reported to be particularly susceptible, leading to a series of abnormalities in the paranodal and internodal regions that caused local axonal swellings and detachment in a mouse model of central fluid percussion mTBI [18]. Microglia and astrocytes become activated by the excess ATP and Ca^2+^ released from damaged axons and neurons within hours following the initial trauma and increase in number and size over the weeks following mTBI, with changes persisting for up to 12 months in the corpus callosum [21,33,34]. Astrocytes can also propagate Ca^2+^ influx into neurons via glial-neuronal signalling pathways, triggering further depolarisation of neuronal membrane and glutamate release [35].

Given the consequences of excess Ca^2+^ entering neurons and glia after injury, the authors have assessed the effects of a combination of ion channel inhibitors (ICI): Lomerizine (Lom), YM872, and Adenosine 5′-triphosphate periodate oxidized sodium salt (oxATP) to inhibit VGCCs, AMPA receptors, and P2X_7_ receptors, respectively. They have shown reductions in excessive Ca^2+^ influx and increased neuronal and glial cell viability in vitro using the ICI combination [36], and reduced oxidative damage, improved myelin structure, and functional recovery following administration of the combinatorial ICI following partial CNS injury in rats [37,38,39]. However, the relative inability of oxATP to cross the blood–brain barrier (BBB) and its toxicity to the cardiovascular system limits the clinical application of this compound [40,41]. As an alternative to oxATP, Brilliant Blue G (BBG) is a highly selective P2X_7_ receptor antagonist with low toxicity [42,43]. Its capacity to cross the BBB makes it an ideal candidate as a clinically relevant drug for protection of neurons and glia. Here, the authors explore the effect of the combinatorial treatment of ICI (Lom, YM872, and BBG) in a rat model of repeated closed-head weight-drop mTBI, assessing behavioural outcomes, cellular responses, oxidative stress, and node of Ranvier integrity.

## 2. Results

In order to assess the effects of combinatorial ICI at 11 days following rmTBI in rats, the current study design incorporated behavioural tests to assess motor function, spatial learning, and memory. Sagittal sections of brain were immunohistochemically analysed for cellular responses, oxidative stress, and integrity of the node of Ranvier.

### 2.1. Behavioural Outcomes

**Figure 1:**
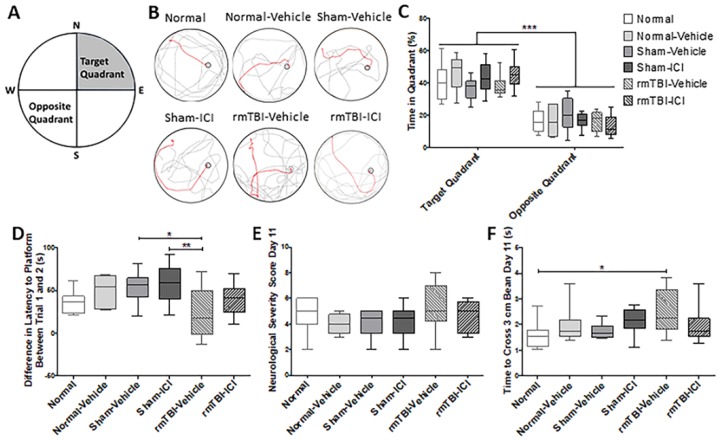
Behavioural outcomes on day 11 following mild traumatic brain injury (mTBI) on days 1 and 2. (**A**) Target and opposite quadrants in the Morris water maze (MWM) testing pool. (**B**) Representative track plots of animal paths in trial 1 (grey) and 2 (red) of the MWM test. (**C**) Box and whisker plots show the median, interquartile range, and range for the time spent in the target and opposite quadrants, (**D**) the difference in latency to the platform between trials 1 and 2, (**E**) the total neurological severity score (NSS) scores, and (**F**) the time to cross the 3 cm beam. * *p* < 0.05, ** *p* < 0.01, *** *p* < 0.001, one-way analysis of variance with Bonferroni post-hoc comparisons.

**Figure 2:**
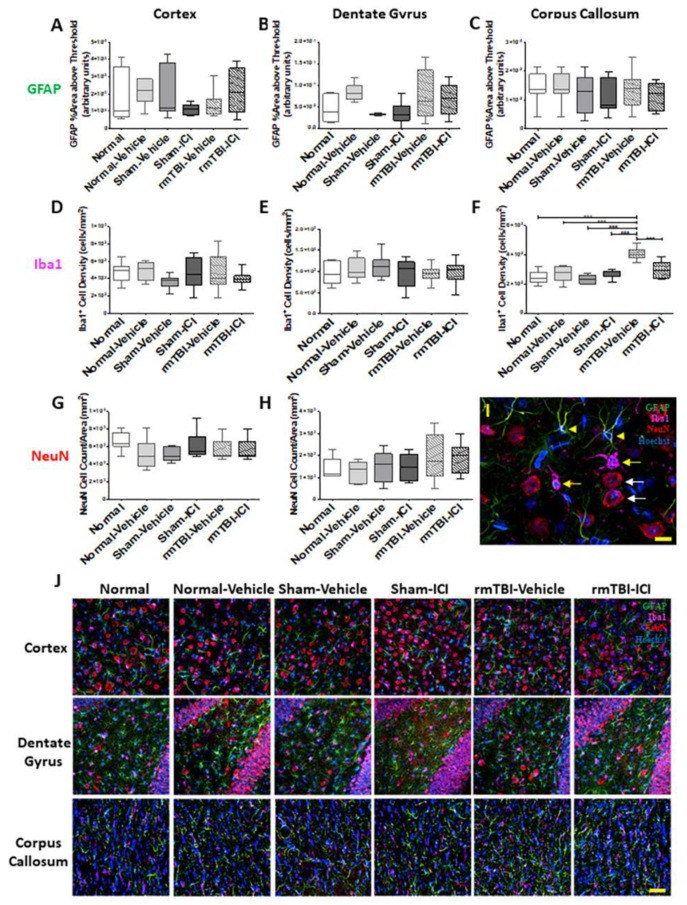
Cellular responses in the brain on day 11 following mTBI on days 1 and 2. (**A**–**C**) Percentage area above threshold of glial fibrillary acidic protein (GFAP) immunoreactivities, (**D**–**F**) Iba1^+^ cell densities, and (**G**–**H**) NeuN^+^ cell densities are shown as median, interquartile range, and range in the middle cortex, the hilus of the dentate gyrus, and the splenium of the corpus callosum, respectively. Neuronal nuclear antigen (NeuN) positive cells were not observed in the corpus callosum. (**I**) Image of immunohistochemical staining at high magnification (600×) shows GFAP^+^ (green) astrocytes (arrow head), Iba1^+^ (magenta) microglia (yellow arrow), and NeuN^+^ (red) neurons (white arrow) co-localised with Hoechst (blue), respectively; scale bar = 20 μm. (**J**) Representative images of GFAP (green), Iba1 (magenta), and NeuN (red) immunohistochemical staining with Hoechst nuclear stain (blue); scale bar = 100 μm. *** *p* < 0.001, one-way analysis of variance with Bonferroni post-hoc comparisons.

The Morris water maze (MWM) test was used to evaluate spatial learning and memory following rmTBI. (Figure 1A). Representative track plots of animal paths demonstrated a similar pattern of pathway to reach the platform across all groups in trial 1 (grey, Figure 1B). The injured group treated with vehicle took a complex pathway to reach the target platform in trial 2 (Red), whereas the rmTBI group with ICI treatment took a more direct pathway to the platform (Figure 2B), similar to normal and sham animals. Quantification showed that all groups spent significantly more time in the target quadrant than the opposite quadrant at day 11 post-injury (*p* < 0.001, F(1,48) = 160.5; Figure 1C). Since the latency to platform improved most quickly between trials 1 and 2 on day 9, the differences between the time to reach the platform in these two trials was evaluated. The time taken to reach the platform in trial 2 was deducted from the time taken for trial 1 for each animal, to calculate the mean improvement between trials. There were significant differences in the difference in time to reach the platform between trials 1 and 2 (*p* = 0.006, F(5,48) = 3.753; Figure 1D). The rmTBI animals administered vehicle demonstrated a significant smaller difference in latency to platform between trials 1 and 2 compared with the two sham groups, thereby showing less improvement in the speed of finding the platform (*p* = 0.039 for rmTBI-Vehicle vs Sham-Vehicle, *p* = 0.009 for rmTBI vs Sham-ICI). The difference in latency to platform between trials 1 and 2 from rmTBI animals treated with ICI was not different to the sham and normal control animals (*p* > 0.999 for all comparisons).

The neurological severity score (NSS) test comprised a series of assessments involving motor, sensorimotor, and vestibulomotor domains to generate total scores ranging from 0 to 15; the higher the score assigned, the greater the dysfunctions observed. There were no significant differences between total NSS scores of any group at day 11 post-injury (*p* = 0.2821, Kruskal–Wallis statistic = 6.257; Figure 1E). The authors have previously observed deficits in the beam-walk element of the NSS [23]. Beam-walk deficits have also been assessed as an isolated assay following repeated mTBI in a range of rodent studies [44,45,46,47]. As such, there is an a priori assumption that the beam-walk outcomes will change, so a sub-analysis of the beam-walk element of the NSS was conducted. Sub-analyses for other NSS outcome measures were not conducted. There were significant differences in the time to cross 3 cm between all experimental groups (*p* = 0.047, F(5,48) = 2.455; Figure 1F). The injured animals with vehicle were significantly slower crossing the 3 cm beam compared with the normal animals (*p* = 0.0382). The injured animals with ICI treatment showed no significant differences in the time to cross a 3 cm beam at day 11 compared with all the sham and normal controls (*p* = 0.723 for Normal, *p* > 0.999 for Normal-Vehicle, *p* = 0.946 for Sham-Vehicle, and *p* = 998 for Sham-ICI).

### 2.2. Cellular Responses

The rmTBI and the ICI treatment did not result in any statistically significant differences in glial fibrillary acidic protein (GFAP) immunoreactivity in either the middle cortex (*p* = 0.273, F(5,43) = 1.321; Figure 2A), the hilus of the dentate gyrus (*p* = 0.168, F(5,47) = 1.642; Figure 2B), or the splenium of the corpus callosum (*p* = 0.774, F(5,43) = 0.5012; Figure 2C). The Iba1^+^ microglia densities were not significantly different between groups in the middle cortex (*p* = 0.594, F(5,45) = 0.7447; Figure 2D), or the hilus of the dentate gyrus (*p* = 0.712, F(5,42) = 0.5838; Figure 2E). However, the densities of ionised calcium binding molecule 1 (Iba1)^+^ microglial cells were significantly different between experimental groups in the splenium of the corpus callosum (*p* < 0.001, F(5,40) = 17.55; Figure 2F). The microglia density in rmTBI animals with vehicle was significantly increased in the splenium of the corpus callosum compared with the sham and normal controls (*p* < 0.0001 for all comparisons). The ICI treatment resulted in a significant decreased density of microglial cells in the splenium of the corpus callosum compared with rmTBI animals administered vehicle (*p* < 0.001). The rmTBI and the ICI treatment did not result in any statistically significant differences in neuronal nuclear antigen (NeuN)^+^ neuron densities in either the middle cortex (*p* = 0.192, F(5,46) = 1.554; Figure 2G) or the hilus of the dentate gyrus (*p* = 0.365, F(5,43) = 3.394; Figure 2H). Densities of neurons were not assessed in the corpus callosum region of the brain due to the absence of NeuN^+^ cells in this region. GFAP^+^ astrocytes (green), Iba1^+^ microglia (magenta), and NeuN^+^ neurons (red) co-localised with Hoechst^+^ nuclei are shown at high magnification in Figure 2I. Representative images of immunohistochemical staining of GFAP, Iba1, and neuronal nuclear antigen (NeuN) for all experimental groups are shown in Figure 2J.

**Figure 3:**
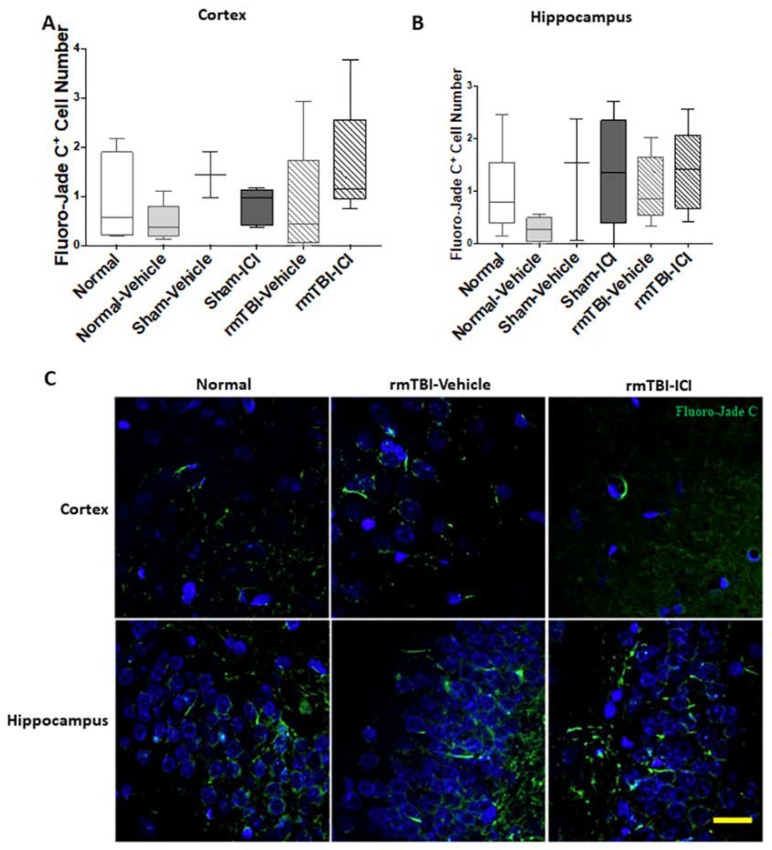
Neuronal degeneration in the brain of day 11 following mTBI on days 1 and 2. Fluoro-Jade C^+^ cell numbers are shown as median, interquartile range, and range in the (**A**) cortex and the (**B**) hippocampus. (**C**) Representative images of Fluoro-Jade C staining indicating degenerating neurons; scale bar = 25 μm.

The rmTBI and the ICI treatment did not result in any statistically significant differences in Fluoro-Jade C^+^ cell numbers in either the cortex (*p* = 0.171, F(5,32) = 1.67; Figure 3A) or the hippocampus (*p* = 0.150, F(5,31) = 1.76; Figure 3B). Representative images of histochemical staining of Fluoro-Jade C are shown in Figure 3C.

### 2.3. Oxidative Stress

**Figure 4:**
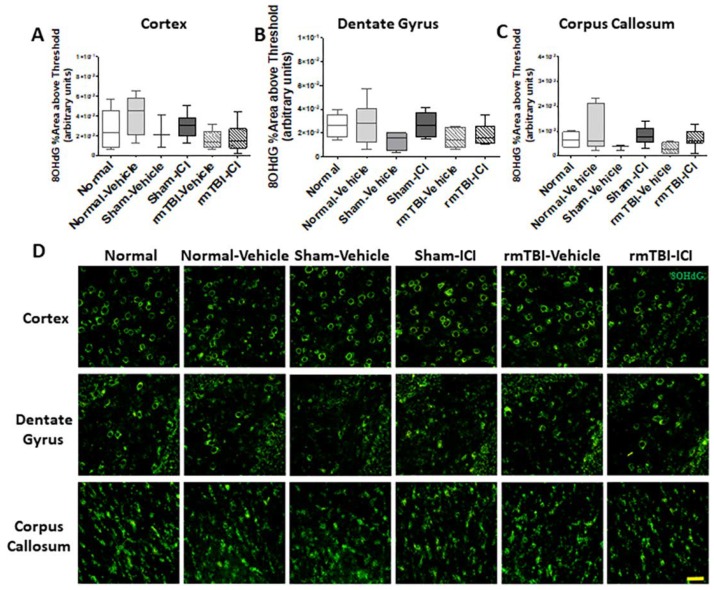
DNA oxidation in the brain on day 11 following mTBI on days 1 and 2. Percentage area above threshold of 8-hydroxy-2′-deoxyguanosine (8OHdG) immunoreactivities are shown as median, interquartile range, and range in the (**A**) middle cortex, the (**B**) hilus of the dentate gyrus, and the (**C**) splenium of the corpus callosum. (**D**) Representative images of 8OHdG (green) immunohistochemical staining indicating DNA oxidation; scale bar = 100 μm.

The immunoreactivity of 8-hydroxy-2′-deoxyguanosine (8OHdG), was assessed as an indicator of oxidative damage to DNA [48]. The rmTBI and the ICI treatment did not result in any statistically significant differences in 8OHdG immunoreactivity in either the middle cortex (*p* = 0.123, F(5,30) = 1.913; Figure 4A), the hilus of the dentate gyrus (*p* = 0.163, F(5,30) = 1.709; Figure 4B), or the splenium of the corpus callosum (*p* = 0.144, F(5,30) = 1.797; Figure 4C). Representative images of 8OHdG immunoreactivity are shown in Figure 4D.

**Figure 5:**
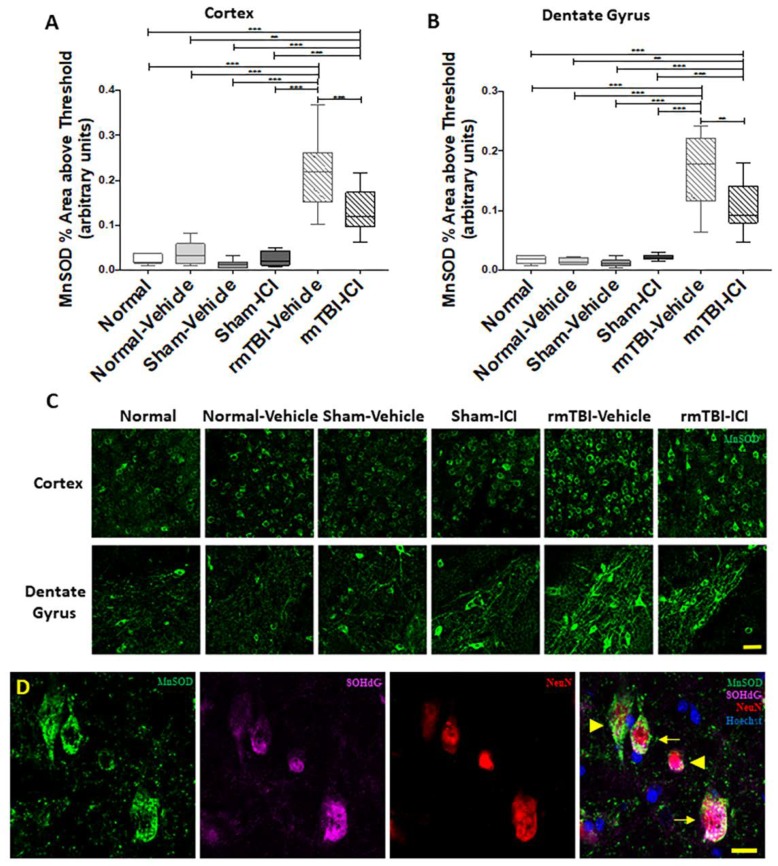
Manganese-dependent superoxide dismutase (MnSOD) immunoreactivity in the brain on day 11 following mTBI on days 1 and 2. Percentage area above threshold of MnSOD immunoreactivities are shown as median, interquartile range, and range in the (**A**) middle cortex and the (**B**) hilus of the dentate gyrus. (**C**) Representative images of MnSOD immunofluorescence (green) indicating oxidative stress; scale bar = 100 μm. MnSOD immunoreactivity was not observed in the corpus callosum. (**D**) High-magnification (1000×) image of MnSOD (green), 8OHdG (magenta), and NeuN (red) immunofluorescence at the middle cortex. Merged image demonstrates co-localisation (arrow) of MnSOD, 8OHdG, and NeuN with Hoechst (blue), or lack or co-localisation of MnSOD with 8OHdG (arrowheads); scale bar = 20 μm. ** *p* < 0.01, *** *p* < 0.001, one-way analysis of variance with Bonferroni post-hoc comparisons.

MnSOD is an antioxidant enzyme which catalytically converts free radical superoxide to hydrogen peroxide and serves as an initial marker for oxidative stress [49,50]. MnSOD immunoreactivity was not observed in the corpus callosum. A semi-quantitative analysis of the MnSOD^+^ area above threshold demonstrated statistically significant differences between experimental groups in the middle cortex (*p* < 0.001, F(5,47) = 32.94; Figure 5A) and the hilus of the dentate gyrus (*p* < 0.001, F(5,45) = 31.84; Figure 5B). The levels of MnSOD immunoreactivity in rmTBI animals administered vehicle were significantly higher than that in sham and normal animals in the middle cortex (*p* < 0.001 for all comparisons) and the hilus of the dentate gyrus (*p* < 0.001 for all comparisons). The ICI treatment led to reduced levels of MnSOD immunoreactivity compared with rmTBI animals with vehicle in the middle cortex (*p* = 0.001) and the hilus of the dentate gyrus (*p* = 0.002). The injured animals receiving ICI treatment also demonstrated a significant increase in MnSOD immunoreactivity compared to all the sham and normal controls in the middle cortex (*p* = 0.001 for Normal, *p* = 0.001 for Normal-Vehicle and *p* < 0.001 for Sham-Vehicle and ICI) and the hilus of the dentate gyrus (*p* < 0.001 for Normal and *p* < 0.001 for all other comparisons). Representative images of MnSOD immunoreactivity are shown in Figure 5C. MnSOD expression was sometimes co-localised with the 8OHdG immunoreactivity in the cytoplasm of NeuN^+^ neuronal cells (arrow, Figure 5D), but not always (arrow heads, Figure 5D).

### 2.4. Node of Ranvier Integrity

**Figure 6:**
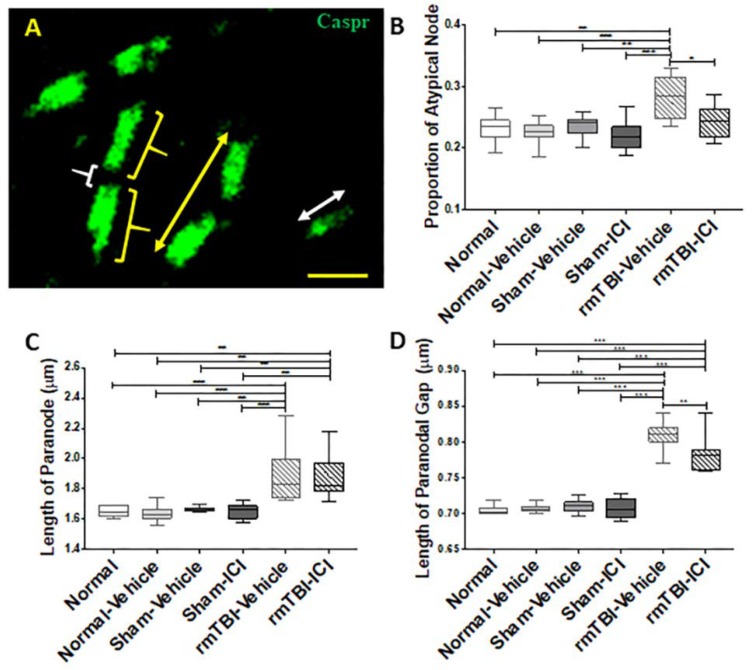
Analysis of the node of Ranvier integrity in the corpus callosum on day 11 following mTBI on days 1 and 2. (**A**) Representative image of Caspr immunofluorescence in the splenium of the corpus callosum shows the measures used in the analysis, including the node of Ranvier complex (yellow arrow), atypical node (white arrow), paranode (yellow bracket), and paranodal gap (white bracket); scale bar = 2 μm. (**B**) Proportion of atypical nodes, (**C**) length of paranode, and (**D**) length of paranodal gap are shown as median, interquartile range, and range. * *p* < 0.05, ** *p* < 0.01, *** *p* < 0.001, one-way analysis of variance with Bonferroni post-hoc comparisons.

Fine-scale analyses of the node of Ranvier integrity in the corpus callosum were conducted using Caspr immunohistochemical identification of paranode structures to reveal subtle changes in structure [51]. A node–paranode complex (yellow arrow) was comprised of a paranodal gap (white bracket) between two paranodes (yellow bracket), indicated by neurexin IV (Caspr) immunoreactivity, whereas an atypical node comprised a single paranode (white arrows; Figure 6A). The proportions of atypical nodes were different between experimental groups (*p* < 0.001, F(5,47) = 7.702; Figure 6B). Post-hoc comparisons revealed a significant increase in the proportion of atypical nodes in the rmTBI animals with vehicle compared to all the control groups (*p* = 0.002 for Normal, *p* < 0.001 for Normal-Vehicle, *p* = 0.007 for Sham-Vehicle and *p* < 0.001 for ICI). The proportion of atypical nodes decreased significantly in the injured animals treated with ICI compared with rmTBI animals with vehicle (*p* = 0.012). There were significant differences in node–paranode structure between experimental groups in the length of the paranode (*p* < 0.001, F(5,47) = 11.01; Figure 6C) and the length of the paranodal gap, equating to the length of the node of Ranvier (*p* < 0.001, F(5,47) = 81,73; Figure 6D). The length of the paranode was increased significantly in rmTBI animals receiving vehicle or ICI treatments compared with controls (rmTBI-Vehicle: *p* = 0.001 for Normal, *p* < 0.001 for Normal-Vehicle, *p* = 0.002 for Sham-Vehicle, and *p* < 0.001 for Sham-ICI; rmTBI-ICI: *p* = 0.003 for Normal, *p* = 0.001 for Normal-Vehicle, *p* = 0.007 for Sham-Vehicle, and *p* = 0.002 for Sham-ICI). However, there were no significant differences in the paranodal length between vehicle- and ICI-treated rmTBI animals (*p* > 0.999). The length of the paranodal gap was also increased significantly in both rmTBI groups compared with controls (*p* < 0.0001 for all comparisons). However, in contrast to the paranode length, the length of the paranodal gap was reduced significantly by the ICI treatment in injured animals, compared with rmTBI animals with vehicle (*p* = 0.001).

## 3. Discussion

The authors have demonstrated that the ICI treatment significantly decreased microglial density in the splenium of the corpus callosum, MnSOD immunoreactivity in the middle cortex and the hilus of the dentate gyrus, and node of Ranvier abnormalities in the splenium of the corpus callosum at 11 days following rmTBI. In the presence of ICI, the improvements in ionic homeostasis may modulate the activation of Na^+^/K^+^-ATPase, resulting in reduced neuronal membrane depolarization and metabolic function disruption. Beneficial effects may be due to reductions in excess intracellular Ca^2+^ within mitochondria, associated with reductions in reactive species, which are elevated soon after a traumatic event to the brain [52,53]. Upregulated MnSOD serves as an initial marker of increased production of the free radical superoxide [49,54] and also indicates an antioxidant defence to inactivate excessive superoxide in cells [55,56]. Excess superoxide damages cellular components such as DNA, when the enzymatic conversion by MnSOD is insufficient [57]. The unchanged 8OHdG immunoreactivity observed in the current injury paradigm indicates that endogenous MnSOD levels elevated with rmTBI were sufficient to limit excess superoxide production and subsequent oxidative DNA damage. Reduced MnSOD with ICI treatment may reflect further reductions in superoxide leading to a reduced MnSOD protective response.

The authors have previously reported [23] that two closed-head weight-drop mTBI resulted in the most distinctive deficits when comparing 1, 2, and 3 mTBI, and thus this injury paradigm was used in the current study to produce subtle impairments in cognitive and locomotor functions. There was a mild deficit in spatial memory and learning functions in rmTBI animals administered vehicle in the MWM test, in line with the learning and memory deficits found in the Barnes maze test in a mouse model of repeated closed-head mTBI [20,21]. This deficit was associated with increased anti-oxidant responses in the hilus of the dentate gyrus in the hippocampus. However, there was no neuronal degeneration observed in this brain region. Impairment in spatial memory in the MWM test with no neuronal cell loss in the hippocampus was also observed in an early study of traumatic brain injury delivered via fluid percussion injury in rats [58]. The excitatory granular neurons in the dentate gyrus are responsible for mediating hippocampal-dependent learning, along with pyramidal cells in the *Cornu Ammonis* areas [59,60,61]; however, these cells were not assessed specifically in the current study. Similarly, a deficit in motor and balancing function was shown in the injured animals administered vehicle during the 3 cm beam walk of the NSS assessments [62,63].

The cognitive and locomotor deficits observed in the vehicle treated rmTBI rats in both tests may be a downstream event of pathological changes in the corpus callosum, which plays a fundamental role in integrating information and mediating complex behaviours [64]. The alteration of microglial and astrocytic responses has been reported in a long-term (6–18 months) study of closed-head rmTBI [21] and short-term studies with severe injury [65] or more impacts [20] in male mice. The authors observed a significant increase in the number of Iba1^+^ microglial cells in the corpus callosum but not in the other regions of the brain examined, whereas the GFAP^+^ astrocyte area remained unchanged, perhaps indicating mild inflammation. The post-injury time point examined in the current study may be too early to detect the subtle changes in astrocytic activation following mild trauma in the brain [21]. The density of Iba1^+^ microglial cells was significantly decreased by ICI treatment in the injured animals, suggesting a regulatory mechanism via the targeted ion channels. It has been reported that astrocytic L-type VGCCs are upregulated and increase secretion of pro-inflammatory cytokines following traumatic injury [66,67]. P2X_7_ receptors on microglial cells become activated by the excessive amount of ATP released from damaged axons and neurons and allow the entry of pro-inflammatory cytokines to regulate microglial activation [68,69,70,71]. Therefore, the inflammatory responses of microglial cells may have been modulated and suppressed via both Lom-mediated VGCC inhibition and BBG-mediated P2X_7_ receptor inhibition.

The integrity of the node of Ranvier is essential for the propagation of action potentials, and abnormalities in its structure are associated with pathological conduction in the CNS [72,73,74]. The authors observed lengthening of nodes and paranodes in the vehicle-treated rmTBI animals, due to paranodal myelin retraction and splitting [18,75,76]. Following CNS injury, the excess influx of Ca^2+^ via AMPA receptors over-activates the neural proteinase enzyme calpain to cleave myelin, leading to paranodal myelin loop eversion and sheath retraction [77,78,79]. The further degradation of myelin can induce degeneration of the node of Ranvier [80,81], illustrated by the observed increase in the proportion of atypical nodes in the corpus callosum in this study. A strong correlation between calpain-related abnormalities in the node of Ranvier and functional deficits has been shown following controlled cortical impact traumatic brain injury [82] and lateral fluid percussion brain injury [83]. In line with the authors’ findings in an alternative partial optic nerve transection in vivo model of neurotrauma [38,39], improvements in the integrity of the node of Ranvier shown as preserved nodal length were seen in the injured animals receiving ICI treatment. This is likely due to antagonism of the Ca^2+^ dependent calpain cleavage of myelin, thereby protecting against paranodal myelin loop eversion and sheath retraction via YM872-mediated AMPA receptor inhibition.

The individual ICI have been well documented separately to limit excess influx of Ca^2+^, oxidative damage, secondary degeneration, and functional deficits in several models of neurotrauma [71,84,85,86,87,88,89,90,91]. However, the assessment of combinations of ICIs for treatment of brain injury has largely been confined to in vitro analyses [92,93], and the examination of the efficacies of individual or combinatorial ICI has not yet been reported in mTBI models. To the authors’ knowledge, this study is the first to report the beneficial effects of the specific combination of ICI (Lom, YM872, and BBG) as a treatment to prevent the damage induced by excess Ca^2+^ influx in TBI of any severity. These results suggest that combinatorial ICI is an attractive therapeutic strategy following rmTBI, modulating antioxidant activity, reducing inflammation, and preserving the integrity of the node of Ranvier. Further studies are required to investigate which modulations of pathophysiology are responsible for the absence of behavioural deficits in the injured animals treated with ICI.

## 4. Materials and Methods

### 4.1. Experimental Model

All experimental procedures were carried out in strict accordance with the Australian Code of Practice for the Care and Use of Animals for Scientific Purposes, National Health and Medical Research Council, and approved by The University of Western Australia Animal Ethics Committee (Approval Number RA/3/100/1366, 20 January 2015–19 January 2020). Adult female Piebald Viral Glaxo rats (160–200 g) obtained from the Animal Resource Centre (Murdoch, WA, Australia), were maintained in standard cages with ad libitum access to food and water on a 12:12 h light–dark cycle. Animals were acclimatised to housing conditions for a minimum of one week prior to any procedures. A total of 54 animals were randomly assigned to one of six experimental groups: normal (*n* = 7), normal treated with vehicle (*n* = 7), sham treated with vehicle (*n* = 8), sham treated with ICI (*n* = 8), rmTBI treated with vehicle (*n* = 12), or rmTBI treated with ICI (*n* = 12).

The procedures to model mTBI were validated and described previously in detail [23]. Briefly, a custom-built weight-drop device (Northeast Biomedical, Tyngsborough, MA, USA), similar to that described in Kane, Angoa-Pérez, Briggs, Viano, Kreipke, and Kuhn [19], was used to deliver rmTBI on days 1 and 2. Animals were anaesthetised with 4% isoflurane in 4 L/min oxygen and maintained at 2% isoflurane in 2 L/min oxygen. A delicate task wiper (Kimwipes, Kimberly-Clark, Irving, TX, USA) was clamped around the edge of a hole on the platform. The animal was placed onto the wiper with the head lined up under the guide tube to ensure the impact site on the midline 2–3 mm anterior to the front of the ears (lambda on the skull). The animal received a 250 g weight released from 1 m height onto the impact site. Analgesia (Carprofen, 4 mg/kg, i.p., Norbrook Laboratories, Tullamarine, VIC, Australia) was administered immediately following the impact, and the animal recovered on a 37 °C heating pad. Sham animals were subjected to identical procedures except for the weight drop, whereas normal animals did not receive anaesthesia or mTBI procedures.

### 4.2. Combinatorial Treatment

Treatment or vehicle administration began after full recovery from anaesthesia on the day of first mTBI, sham, or equivalent time in normal animals (day 1). The dosing regimens of ICI were based on previously published studies showing efficacy using these agents individually. Lom (30 mg/kg; LKT Laboratories, St Paul, MN, USA) was administered orally twice daily in butter vehicle until euthanasia on day 11 [94,95]. YM872 (20 mg/kg; LKT Laboratories, St Paul, MN, USA) and BBG (50 mg/kg; Sigma-Aldrich, St Louis, MO, USA) were dissolved in 1 mL sterile phosphate buffer saline (PBS) vehicle and delivered intraperitoneally (i.p.) every 48 h until euthanasia [86,89].

### 4.3. Behavioural Assessments

Animals were assessed for spatial learning and memory functions by MWM between days 9 and 11, and neurological deficits using the NSS at days 2, 5, 8, and 11. Rats were not habituated to either testing apparatus prior to testing.

The NSS assessment was modified from Chen, Constantini, Trembovler, Weinstock, and Shohami [62] and Stahel, Shohami, Younis, Kariya, Otto, Lenzlinger, Grosjean, Eugster, Trentz, and Kossmann [63] to increase the sensitivity for rats. Performance of reflexes and motor abilities was assessed through a 15-task checklist, including ability to move, presence of righting reflex, ability to walk in a straight line, hemiplegia and monoplegia, flexion of hind limbs when raised by the tail, startle reflex, seeking behaviour, prostration, placing reflexes for each limb, ability to stay on 2 × 2 cm and 5 × 5 cm platforms 30 cm above ground for 1 min, ability to balance on a 1 cm round beam for 1 min and ability to cross 1 cm, 2 cm, and 3 cm beams without foot faulting. There were multiple assessing items in each task and each assessing item was given a binary score of 1 for a failure or 0 for a success. The scoring system allowed for a maximum possible score of 22, representing the most severe neurological dysfunction. Investigators conducting NSS analyses were not blinded to group identity.

MWM: a 1.65 m diameter pool was filled with water to 30 cm depth and made opaque by the addition of non-toxic white poster paint (Crayola, Easton, PA, USA). Water was heated to 24–26 °C with an aquarium heater. The quadrants of the pool were labelled N, S, W, and E. Each wall of the room contained a visual cue which may have been used to aid orientation. Testing followed established procedures [96] and took place over 3 consecutive days: days 9 (acquisition), 10 (reversal), and 11 (probe). On the acquisition and reversal days, there was a 10 cm round platform placed in the SW (opposite) and NE (target) quadrants, respectively, which were covered by 1–2 cm of water to obscure it from view. Rats were placed in the water facing the wall and allowed to swim until they found the hidden 10 cm platform. If 2 min passed without the rat finding the platform, they were placed on it for 15 s before drying under a 50 W heating lamp. There were four trials for each rat in pseudorandom order, with one trial in each quadrant and an interval of 10 min between each trial. On the probe day, the platform was removed and the animals were allowed to swim freely for 90 s. A webcam (C270, Logitech, Lausanne, Switzerland) was mounted on the ceiling for video recording and the video was analysed by investigators who were blind to group identity, using tracking software ANY-Maze (version 4.99, Stoelting, IL, USA).

### 4.4. Immunohistochemistry

At day 11, the animals were euthanised with Lethabarb (sodium pentobarbital, 100 mg/kg, i.p.; Virbac, Australia), and perfused transcardially with saline (0.9% sodium chloride) followed by 4% paraformaldehyde (VWR Chemicals, Radnor, PA, USA) in phosphate buffer (0.1 M, pH 7.2). Brains were dissected and post-fixed in 4% paraformaldehyde (VWR Chemicals, Radnor, PA, USA) overnight, and then cryoprotected in 15% sucrose in PBS with 0.1% sodium azide (Sigma-Aldrich, St Louis, MO USA) until cryosectioning. Sagittal sections (20 μm) were mounted onto Superfrost Plus slides (Thermo Fisher, Waltham, MA, USA) and air dried for one hour for immunohistochemical and histochemical stainings. Slides were washed in PBS for 2 × 5 min followed by blocking solution containing PBS with 0.2% Triton-X 100, 1% bovine serum albumin, and 5% normal donkey serum for 10 min. Sections were then incubated overnight at 4 °C in blocking solution containing primary antibodies: goat anti-Iba1 (1:1000, Abcam, Cambridge, UK, RRID: AB_2224402) to label microglia; rabbit anti-GFAP (1:1000, Agilent Technologies, Santa Clara, CA, USA, RRID: AB_100130082) to label astrocytes; mouse anti-NeuN (1:400, Novus, Littleton, CO, USA, RRID: AB_11023082) to label neurons; mouse anti-Caspr (1:500, NeuroMab, Davis, CA, USA, RRID: AB_2083496) to label paranodes; mouse anti-8OHdG (1:500, Abcam, Cambridge, UK, RRID: AB_940049) to detect oxidized DNA and rabbit anti-MnSOD (1:500, Enzo Life Sciences, Farmingdale, NY, USA, RRID: AB_2051889) to indicate endogenous antioxidant responses. On the following day, all the slides were washed with PBS for 3 × 5 min. Sections were incubated for 2 h at room temperature with appropriate species/isotype-specific secondary antibodies (AlexaFluor 488, 555, or 647, 1:400, Thermo Fisher, Waltham, MA, USA) in combination with Hoechst nuclear stain (0.01 µg/mL, Thermo Fisher, Waltham, MA, USA). All slides were washed in PBS for 3 × 5 min and cover-slipped with Fluoromount G (Thermo Fisher, Waltham, MA, USA).

Degenerating neurons were visualized by histochemical staining of Fluoro-Jade C according to the manufacturer’s instructions (Biosensis, Adelaide, SA, Australia). Briefly, slides were dried at 60 °C for 10 min, and incubated in sodium hydroxide for 5 min and in 70% ethanol for 2 min. All slides were incubated with 0.06% potassium permanganate for 10 min and washed with distilled water for 2 min. They were then incubated with 0.0001% Fluoro-Jade C in 0.1% acetic acid for 10 min. All slides were washed in distilled water for 3 × 2 min, air dried, cleared in xylene and cover-slipped with Entellan NEW (Merck Millipore, Burlington, MA, USA).

### 4.5. Microscopy and Image Analysis

Imaging for quantification was conducted using a Nikon Ti-E inverted microscope, controlled by NIS elements software (version 4.0, Nikon Instruments, Melville, NY, USA) or a Nikon C2 mounted Ni-E upright confocal microscope, controlled by NIS elements software (version 4.3, Nikon Instruments, Melville, NY, USA). Brain regions of interest selected for immunohistochemistry imaging were the middle cortex, defined as the dorsal area above the hippocampus, the hilus of the dentate gyrus in the hippocampus, and the splenium of the corpus callosum. Immunohistochemistry images were captured in 6 μm z-stacks at 0.5 μm optical thickness deconvoluted using NIS elements software (version 4.0, Nikon Instruments, Melville, NY, USA). Quantification of Fluoro-Jade C staining was conducted in the entire cortex and all regions of the hippocampus. Fluoro-Jade C staining images were captured in 15 z-stacks at 1 μm optical thickness with PerkinElmer Ultraview Vox coupled with Volocity imaging software, and Fluoro-Jade C positive neurons were manually counted.

Investigators were blinded to group identity for analysing images. Cell density and immunoreactivity analyses were performed on a single image per brain region per animal, normalized for area and background. Sagittal sections closest to the midline were used for quantification to ensure a consistent location and to minimize variability. Image analyses were conducted to determine the area above the threshold intensity using Fiji analysis software (version 2.0.0, National Institute of Health, Bethesda, MD, USA), setting arbitrary threshold intensities normalised to the background. For node–paranode analyses, the lengths of the node of Ranvier and paranode and the proportion of atypical nodes relative to the number of total node–paranode complexes were measured in the splenium of the corpus callosum. One hundred node–paranode complexes in the central 125 × 125 μm area of the field of view were randomly selected for quantification, if they were clearly within 6 μm z-stacks at 0.5 μm optical thickness, as per previously published techniques [51].

### 4.6. Statistical Analysis

All statistical analyses were performed using SPSS Statistics software (version 25, IBM, Armonk, NY, USA), whereas graphs were generated using Prism 7 (version 7.02, GraphPad, La Jolla, CA, USA). All data were expressed as median, interquartile range, and range. One-way analyses of variance with Bonferroni post-hoc tests were used to determine significant differences for normally distributed parametric data: all groups were compared to each other in post-hoc analyses in SPSS. Non-normally distributed data were analysed using the Kruskal–Wallis rank sum test. Differences were considered statistically significant at *p* < 0.05.

## Abbreviations

8OHdG	8-hydroxy-2′-deoxyguanosine
AMPA	α-amino-3-hydroxy-5-methyl-4-isoxazolepropionic acid
BBB	Blood–brain barrier
BBG	Brilliant Blue G
Caspr	Neurexin IV
CNS	Central nervous system
GFAP	Glial fibrillary acidic protein
Iba1	Ionised calcium binding molecule 1
ICI	Ion channel inhibitors
MnSOD	Manganese superoxide dismutase
MWM	Morris water maze
mTBI	Mild traumatic brain injury
Na+/K+-ATPase	Sodium- and potassium-activated adenosine 5′-triphosphatase
NeuN	Neuronal nuclear antigen
NMDA	*N*-methyl-d-aspartate
NSS	Neurological severity score
oxATP	5′-triphosphate periodate oxidized sodium salt
TBI	Traumatic brain injury
rmTBI	Repeated mild traumatic brain injury
VGCC	Voltage-gated calcium channels
